# Influence of stacking sequences of woven jute-carbon hybrid composites: Diffusion mechanism and mechanical characterization

**DOI:** 10.1016/j.heliyon.2024.e36632

**Published:** 2024-08-22

**Authors:** Md Sadbin Azad Alvy, Md Foisal Hossain, Muhammed Sohel Rana, Md Motinur Rahman, Md Shafiul Ferdous

**Affiliations:** a Department of Materials Science and Engineering, Khulna University of Engineering & Technology, Khulna, 9203, Bangladesh; b Department of Electronics and Communication Engineering, Khulna University of Engineering & Technology, Khulna, 9203, Bangladesh; c Central Engineering Facilities, Atomic Energy Research Establishment, Savar, Dhaka, 1349, Bangladesh; d Institute of Electronics, Bangladesh Atomic Energy Commission, Dhaka, 1349, Bangladesh; e Former Faculty, Department of Mechanical Engineering, Khulna University of Engineering & Technology, Khulna, 9203, Bangladesh

**Keywords:** Composites, Diffusion mechanism, Tensile strength, Flexural strength, *FEM*, Corrosive impact

## Abstract

In this study, different stacking orders of carbon-jute fiber mats were used to fabricate the composites and hybrids. The impact of different stacking orientations on mechanical properties was investigated experimentally. The experimental results were verified by finite element analysis (*FEA*). In addition, the impact of the fiber orientation on the loading direction was also investigated. Four layers of fabrics were piled to fabricate the composites by hand-layup with the cold press process. A NaCl solution was used to create a corrosive medium (pH 8), and the composites and hybrids were immersed in that medium, and their mechanical properties were compared. In this research, it has been found that the laminates with the stacking sequence of type-*C* (*C/J/J/C*) show the best result among other composites. Jute composites where all the fibers are in the loading direction possess a high strain compared with the other composites and hybrids. On the scanning electron microscope (*SEM*) image, delamination, buckling of fiber, fiber pullout, and voids were observed. In addition, the kinetics of the diffusion mechanism were also investigated, and it was found from the experimental results that the composites and hybrids immersed in pH 8 absorbed water via Fickian diffusion.

## 1 Introduction

Natural and environmentally friendly materials are gaining popularity nowadays. There is a need to reduce the price of synthetic fibers, which are frequently used in the manufacture of polymer composites. Ecological and environmental concerns have spurred a growing interest in the utilization of natural materials. As a result, lignocellulosic fibers such as flax, jute, hemp, sisal, kenaf, bamboo, and ramie have emerged as a promising and environmentally friendly alternative to glass fibers for reinforcement in engineering composites. Compared to other synthetic fibers, jute fibers have more advantages, such as less toxicity, 100 % biodegradability, less energy consumption, and carbon dioxide neutrality [1]. Jute fiber is a natural fiber that contains hydrophilic polar groups, facilitating the formation of hydrogen bonds with water molecules. When considering the tribological application, the jute fiber showed superior properties than that of neat and glass-reinforced epoxy composites [2]. However, the water absorption capacity of jute may result in poor interfacial bonding between fibers and matrix, which can lead to inferior mechanical properties of natural fiber-reinforced composites. To address this issue, hybrid composites have been proposed [3]. The mechanical and fatigue behaviors of jute composites highlight their slower degradation compared to carbon fiber composites due to their high strain and semi-brittle nature [4]. Synthetic (Kevlar) fiber addition enhanced the load-bearing capacity of the jute fiber composite and its ability to withstand bending strength. Synthetic fiber with jute reduces water sorption [5,6]. A significant number of researchers are working on plain, twill, or basket-type natural fiber mats [[7], [8], [9]]. An investigation of the compression behavior of circular honeycomb sandwich panels fabricated using jute fibers explored the influence of jute fiber orientation on the load-bearing capacity [10]. In addition to being more environmentally friendly than pure carbon fiber composites, the resultant composite outperforms jute fiber composites in terms of mechanical qualities. The combination of low-cost natural fibers, such as jute, with carbon fibers presents a more sustainable alternative to traditional carbon fiber-reinforced polymer (*CFRP*) structures. These have the potential to replace *CFRP* in structures that are susceptible to vibrations [11,12]. The strength of fiber-reinforced composites depends on various factors, including the properties of the fiber, matrix, and interfacial interaction between them. Similarly, as the proportion of jute fiber layers increases, a decrease in flexural and impact strength can be expected [13]. Soliman et al. conducted an experimental and numerical study to determine the on-axis and off-axis flexural strengths of carbon-woven composites. The results showed that the on-axis flexural performance was dominated by the fibers, while the matrix governed the off-axis flexural strength [14]. Abhishek et al. found that composites with a thickness of 3.48 mm and a fiber volume fraction of 15.62 % exhibited the maximum flexural strength [15]. Hongxiao et al. investigated the design optimization of *CFRP* stacking sequences to improve impact strength using a multi-island genetic algorithm. The optimized specimen comprised a variety of angled plies and exhibited 42.1 % less damage than the baseline laminate [16]. Gopinath et al. evaluated the impact strength improved upon fiber treatment with 5 % NaOH [17]. Using carbon nanotube fillers reduced flexural strength due to the agglomeration of filler in composites [18]. While continuous, unidirectional carbon fibers are not given priority in hybrids yet; natural fiber composites are also a possible candidate to be used as biomaterials. The use of synthetic products may result in an increased level of pollution, affecting both the environment and living organisms [19,20]. Natural fiber reinforced composites find increased attention in construction, automotive, furniture, and packaging applications [21]. It can be postulated that hybrid fiber-reinforced composite structures with stiffer plies placed on the exterior will exhibit higher flexural and impact strength [11].

The goal of this study is to examine the mechanical characteristics of woven unidirectional carbon/jute fabric-reinforced epoxy hybrid composites and discover new uses for them in a range of industries, including the construction, automotive, and aerospace sectors. Prior researchers have worked on carbon with jute in various ways, but the unidirectional fiber properties set this work apart from them. In addition, the diffusion mechanism for these specific types of hybrids has yet to be discussed. These types of hybrids also lacked *FEA* comparisons in previous works. In addition, the impact of aging in a corrosive environment (pH 8) on mechanical properties and the diffusion mechanism were also investigated. The resultant hybrids from this research can be used in automotive sectors, reducing the cost of carbon composites. As well as increasing the usage of jute fibers. Hybrids for example, can be used for supercar bodies with light weight and also on side view mirrors that are easily replaceable and feasible [22]. Further research can be conducted on the hybrids to check for biocompatibility as well as prosthetics.

## 2 Experimental procedure

### 2.1 Materials

In this study, untreated jute fiber yarn was used to weave the unidirectional mat on a custom-designed handloom. The areal density of those woven mats was 650 gm per square meter (*GSM*). The diameter of the jute fiber yarn was 1.00 mm. The unidirectional woven carbon fiber mat of 317 *GSM* was obtained from Lab Cast Co. Ltd. in Japan. Epoxy resin (Lapox B-11) and hardener (Lapox K6) were used as matrix for those composites. The properties of the single jute fiber yarn, carbon fiber thread, and matrix used in this study were evaluated and are shown in Table 1.

**Table 1:** The properties of the single fiber yarns and matrix used.

Property	Jute	Carbon	Matrix
Density (g/cm^3^)	1.235	1.79	1.19
Tensile Strength (MPa)	377	5017	22.76
Young's Modulus (GPa)	16.90	238	4.44
Peak strain (%)	2.27	2.10	1.24

### 2.2 Fabrication of composites and hybrids

Hand-layup with a cold press process was used to fabricate the composites and hybrids. The fabrication process involved impregnating the fiber layers with an unsaturated resin that was prepared by mixing the resin hardener at a weight ratio of 11:1 [11,23,24]. To ensure an even distribution of the impregnating resin throughout the reinforcement mats, a hand roller was used. It should be highlighted that all the reinforcement fiber layers in the fabricated composites were oriented in the same direction. The fabricated composites were cured between 75-kg surface plates for a duration of 48 h in room temperature (30 ± 2 °C). It is worth noting that composites with a high fiber-matrix ratio were fabricated in this experiment. Additionally, it is important to note that the direction of the reinforcements was aligned with the loading direction during the experimental tests. Furthermore, two types of jute composites were fabricated with different fiber orientations (45° and 90°) to investigate the impact of fiber orientation towards loading direction on mechanical properties both in base and in pH 8 conditions. The dry specimens are considered ‘base’ specimens. The types of composites fabricated and tested are presented in Table 2.

**Table 2:**
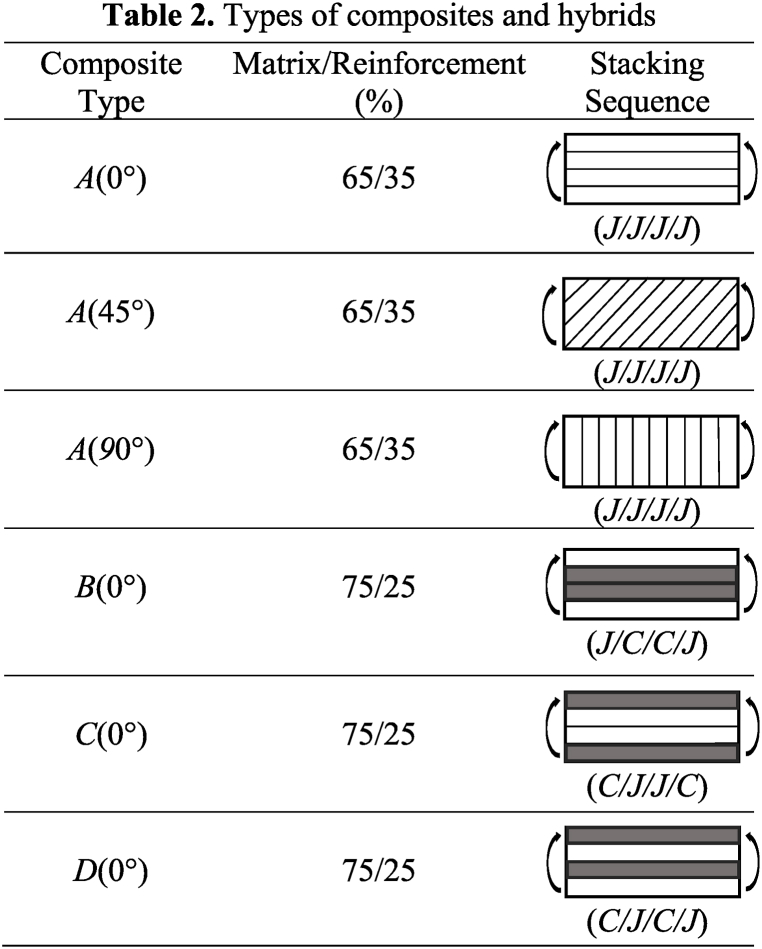
Types of composites and hybrids.

*J* = Jute fiber unidirectional mat; *C* = Carbon fiber unidirectional mat.

### 2.3 Investigation of tensile and flexural strength

In this study, the tensile and flexural properties of both the base and the samples immersed in pH 8 were evaluated in room temperature (30 ± 2 °C). The samples were immersed in NaCl solution (pH 8) for fifteen weeks to investigate the impact of strength on corrosive mediums. At least three specimens of each type were tested. The tensile tests were conducted using a “Testometric M500-100CT” machine with a crosshead speed of 30 mm/min and a pretension of 0.001N. The tensile samples were prepared following the ASTM D638-01 standard [25]. The flexural tests were performed using a Tinius Olsen 25ST machine equipped with a 25 kN load cell and an 80 mm span distance. The flexural samples were prepared according to the ASTM D790 standard [25].

### 2.4 Numerical analysis

A finite element analysis was performed with ABAQUS 6.14-2 to accurately predict the tensile and flexural strengths of both the base samples and the samples in corrosive conditions, as it is one of the most effective and time-saving tools for analyzing elastic behavior. The dimensions of the specimens were coordinated with the experimental samples. The results were then compared with the experimental data.

### 2.5 Impact of water and diffusion mechanism

Water absorption was assessed in jute and jute/carbon hybrid composites. Tensile and flexural samples were machined to dimensions according to ASTM D638-01 and ASTM D790 standards, respectively. The specimens were immerged in a 3 % NaCl solution until they reached the saturation level of water absorption and swelling thickness. After wiping off the water on the surface of the samples, the samples were weighed to the nearest 0.1 mg within 1 min. The moisture content (percent weight expansion) was estimated using the following Eq. (1) [26].

(1) $$W_{m}=\frac{W_{t}-\;W_{0}}{W_{0}}\;X\;100\%$$

where *w*_t_ and *w*_0_ are the weight over time and initial weight, respectively. The change in thickness due to moisture absorption was calculated using the following Eq. (2) [25].

(2) $$h_{m}=\frac{h_{t}-\;h_{0}}{h_{0}}\;X\;100\%$$

where *h*_t_ and *h*_0_ are the thickness over time and the initial thickness, respectively. The Fickian diffusion coefficient (*D*) was calculated using Eq. (3) [25].

(3) $$D=\frac{\pi }{t}{\left(\frac{M_{t}h}{4M_{s}}\right)}^{2}$$

where *M*_s_ is the maximum moisture content at saturation, *h* is the thickness of the corresponding composite, and *M*_*t*_ denotes the water absorption rate at time *t.*

To take into consideration the edge effects, a correction factor is still required, which defines the resultant adjusted diffusion coefficient (*D*_c_), defined by Eq. (5) [26,27].

(4) $$D_{c}=D{\left(1+\frac{h}{l}+\frac{h}{n}\right)}^{-2}$$

where *h*, *n,* and *l* are the thickness, width, and length of the corresponding machined flexural sample, respectively.

### 2.6 SEM analysis

Scanning electron microscopy (*SEM*) was used to observe the surface morphology and fiber/matrix interaction of the fabricated composites and hybrids in both base and samples immersed in pH 8 conditions. A TESCAN VEGA3 scanning electron microscope was used to observe the surface morphology.

### 2.7 Density and void content

The void content of composites can be determined by utilizing the ASTM D-2734-70 [[25], [26], [27]] standard, which involves the calculation of void percentage based on both the theoretical density ($\rho_{t}$) and the actual density ($\rho_{a}$). In order to ascertain the void percentage in the composites, the following formula is used as described in equation (5),

(5) $$V_{v}\;\left(\%\right)=\frac{\rho_{t}-\rho_{a}}{\rho_{t}}\times 100\%$$

Correspondingly, theoretical density $\rho_{t}$ can be determined using equation (6), no matter the types of fibers used to fabricate a composite [25].

(6) $$\rho_{t}=\frac{1}{\left(\sum_{i=1}^{n}\frac{W_{fi}}{\rho_{fi}}+\frac{W_{m}}{\rho_{m}}\right)}$$

where the weight fraction of fiber and matrix is $W_{f}$ and $W_{m}$, respectively. On the contrary, the density of fiber and matrix is denoted by $\rho_{f}$ and $\rho_{m}$, respectively.

The water immersion method was employed to ascertain the actual density. Equation (7) describes the actual density, where in $\rho_{a}$ signifies the actual density and $S_{p}$ stands for the specific gravity of the composites and hybrids [28].

(7) $$\rho_{a}=S_{p}\times 0.9976\;\text{gm}/{\text{cm}}^{3}$$

## 3 Results and discussion

### 3.1 Physical properties

The physical properties and Young's modulus of the fabricated jute/carbon hybrid composites are illustrated in Table 3. It is evident from the results that the Young's modulus of the jute/carbon composites increased approximately twofold in comparison to the jute composite. This improvement in Young's modulus is significant and in agreement with previous research studies [3,4]. However, it should be noted that the value of Young's modulus varied with changes in the stacking sequence. The addition of carbon fibers along with jute fibers in the composites resulted in changes in density and specific gravity. Furthermore, the aerial density decreased with the introduction of carbon fibers in the composites. In addition, the percentage of elongation increased by approximately 2.5 times in comparison to the traditional jute composite of type *A*(0°), with carbon fiber playing a crucial role in this enhancement [3]. As carbon fiber is stronger and stiffer than any other fiber, its mechanical and physical properties increase when hybridize with other fibers. Table 1 shows the experimental results of the physical and mechanical properties of the single yarns and matrix used in this study. Nayeem et al. worked with JUCO fiber mats, incorporated woven carbon fiber mats to fabricate JUCO/carbon hybrid composites. It was proven that by gradually increasing the carbon fiber mats with JUCO fiber, the mechanical and physical properties increased significantly because of the stiff and strong nature of carbon fibers [29]. These results demonstrate the potential for improving the mechanical properties of jute composites by incorporating carbon fibers [30].

**Table 3:** Physical properties and Young's modulus of composites and hybrids.

Composite Type	Density, $\rho a\left(kg/m^{3}\right)$	Specific Gravity	% of Elongation	Young's Modulus	Aerial Density, (*GSM*)
*A*(0°)	1050	1.05	0.8818	15596	7927
*A*(45°)	1070	1.07	0.4852	8939	7556
*A*(90°)	1140	1.14	0.4100	5330	7900
*B*(0°)	1154	1.16	2.0386	33812	5770
*C*(0°)	1099	1.10	2.2432	30600	4950
*D*(0°)	1144	1.15	2.1158	27569	4869

### 3.2 Tensile test

**Fig. 1:**
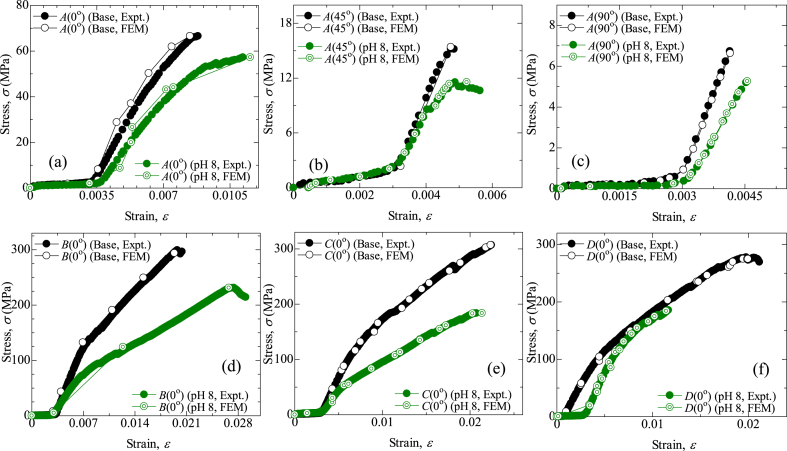
Comparison of stress vs. strain curve of base and aging by pH 8: (a) *A*(0°) type, (b) *A*(45°) type, (c) *A*(90°) type, (d) *B*(0°) type, (e) *C*(0°) type, (f) *D*(0°) type.

The effect of hybridization on the tensile behavior of jute composites and jute-carbon hybrids, as well as different stacking sequences, is presented in Fig. 1. From the experimental and *FEA*, it was proven that fiber orientations among *A* types have a significant impact on the mechanical properties of the fabricated composites. Type-*A*(90°) exhibits the lowest tensile strength, and type-*A*(0°) showed the highest strength among *A*-type composites. Type-*A*(0°) having the same fibers direction as the tensile loading direction, essentially elevated this type of strength [25]. Hybridization with carbon fiber, the tensile strength increased up to 4.5 times. The type-*C*(0°) shows the maximum tensile strength (306.87 MPa) where all the carbon fiber mats cover jute fiber mats (*C/J/J/C*). The introduction of unidirectional woven carbon fibers increased the tensile value up to 361 % in (*C/J/J/C*), compared to the strength of the *A*(0°) type specimen. However, *A*(0°) shows the maximum strain due to the jute strain compatibility. Suhad et al. also showed that jute composites possess high strain compared to pure carbon composites, which gives an overall improvement in mechanical behavior [4,31].

**Fig. 2:**
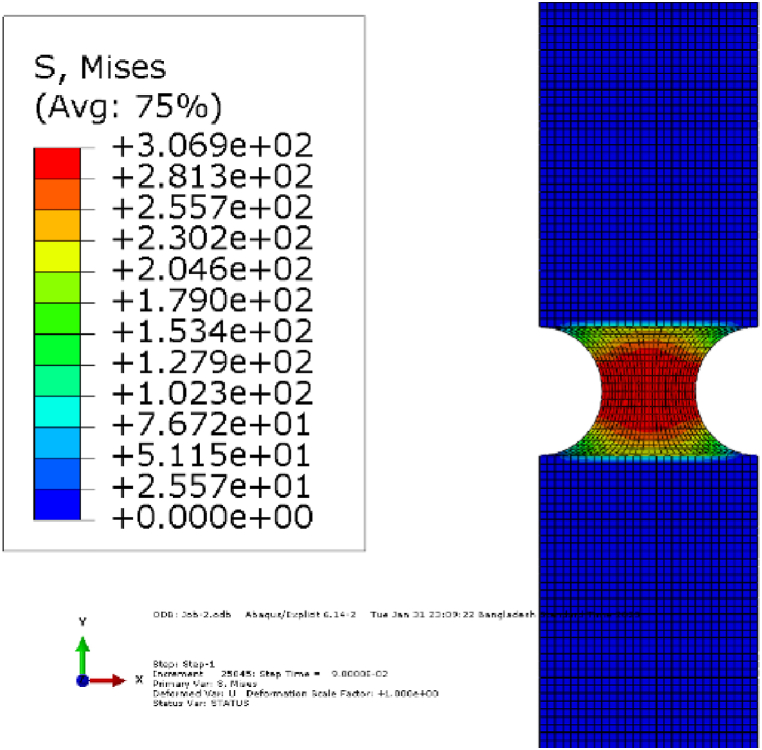
Contour plots for von-Mises stress for tensile simulation (base) of type-*C*(0°).

**Fig. 3:**
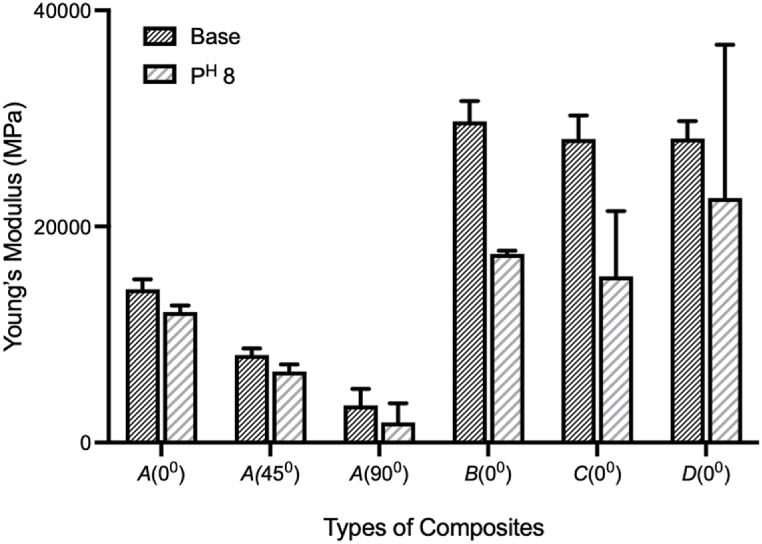
Comparison of Young's modulus of elasticity (Expt.) in different condition. Data shown as mean ± SE.

In addition, the impact of water aging under corrosiveness was also illustrated in Fig. 1(a–f). The immersion medium was inspected regularly to ensure constant pH levels. The tensile strength decreased in all cases of composites and hybrids, despite increased strain. There are various reasons why the tensile strength and strain of carbon/jute hybrid composites vary between base and post-corrosion tests. Immersion in a 3 % NaCl solution could lead to water uptake by the fibers due to its hydrophilic property, which can weaken the composite bonding between the matrix and fiber, reduce its load-carrying capacity, and increase deformation. This could be due to several reasons, like the presence of voids, porosity, or defects within the composite that allowed the water solution to penetrate and form a micro-crack within the composite [25,[32], [33], [34], [35]]. Corrosion also reduces the strength of the fibers themselves, which further reduces total strength [[36], [37], [38]]. Carbon fiber layers in both surfaces covering inner jute layers significantly reduce water absorption and corrosion, which is also shown in *SEM* analysis in Fig. 8(c). Similar results were seen when using Kevlar and jute hybrids [25]. In Fig. 2, base specimens (type-*C*(0°)) tensile stress contour plots of different sequences are presented. Fig. 3 illustrates the comparison of Young's modulus of elasticity in different conditions. Corrosion reduced the Young's modulus values for all types. The degradation in Young's modulus could be attributed to the water penetration within the fiber layers [36]. Table 4 shows the summary of tensile strength by experimental and *FEM* of base and in pH 8 condition with relative mismatch or deviation.

**Table 4:** Summary of tensile strength of base samples and in pH 8 condition.

Composite Type	*σ*_u_ (Expt.) Base (MPa)	*σ*_u_ (*FEM*) Base (MPa)	Deviation (%)	*σ*_u_ (Expt.) pH8 (MPa)	*σ*_u_ (*FEM)* pH8 (MPa)	Deviation (%)
*A*(0°)	66.54	66.54	0	57.24	57.24	0
*A*(45°)	15.18	15.40	1.45	11.54	11.54	0
*A*(90°)	6.74	6.74	0	5.20	5.20	0
*B*(0°)	296.50	296.5	0	231.17	231.17	0
*C*(0°)	306.88	306.88	0	183.69	183.69	0
*D*(0°)	277.70	274.68	1.09	185.65	185.65	0

### 3.3 Flexural test

**Fig. 4:**
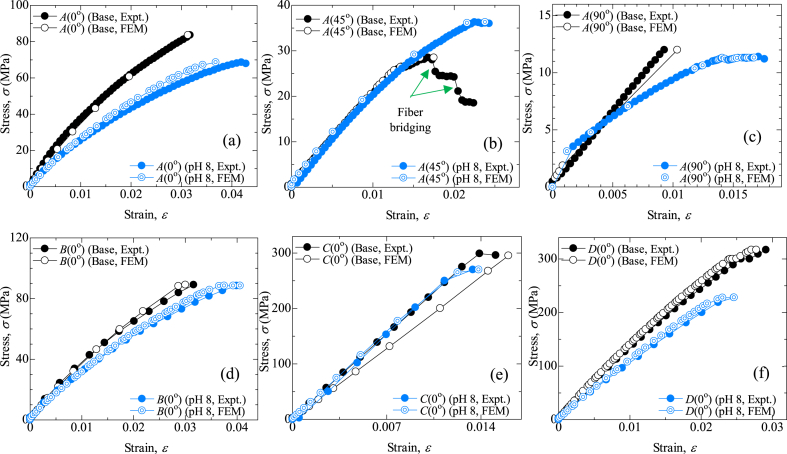
Comparison of flexural stress vs. strain curve of base and aging by pH 8: (a) *A*(0°) type, (b) *A*(45°) type, (c) *A*(90°) type, (d) *B*(0°) type, (e) *C*(0°) type, (f) *D*(0°) type.

**Fig. 5:**
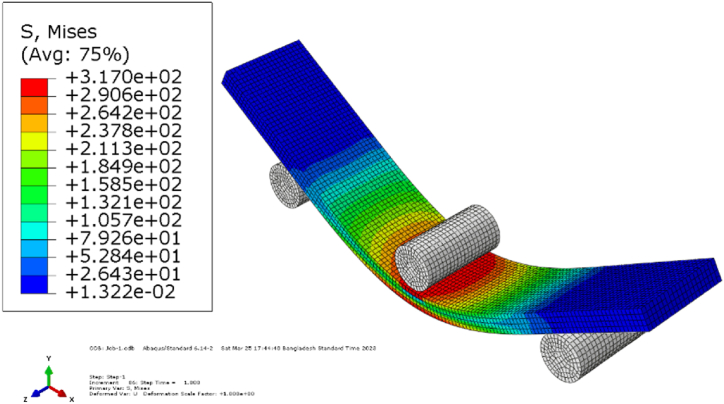
Contour plots of von-Mises stress for flexural simulation (base) of type-*D*(0°).

**Fig. 6:**
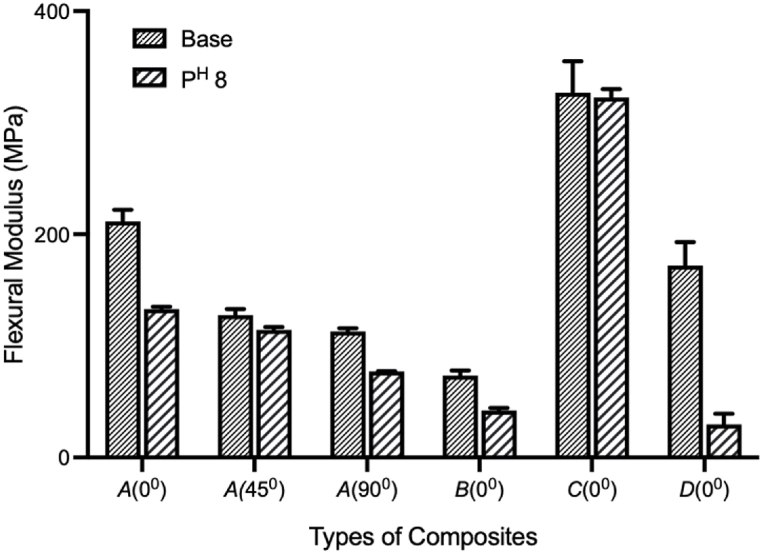
Comparison of the flexural modulus of elasticity (Expt.) in different conditions. Data shown as mean ± SE.

Fig. 4(a–f) shows the experimental and *FE*-simulated flexural stress-strain diagrams of the laminates. Almost similar behavior was found in flexural strength except for type-*A*(45°). The flexural strength decreased when the samples were immersed in pH 8 mediums. However, due to the fiber bridging of base sample in type *A*(45°), reverse situation occurred. The carbon fiber outer layers in types *C*(0°) and *D*(0°) show higher flexural strength due to the bending superiority of carbon fibers. Type *C*(0°), showed about a 258 % increase over type *A*(0°). While type *D*(0°) flexural strength increased by about 280 %. All these values establish the superiority of hybrid composites over jute fiber composites [30,31,39]. As jute mats are in between the carbon mats in *C*(0°) type specimen, water can hardly penetrate inside the jute mat, which is described in the impact of the water absorption section. Therefore, *C*(0°) type specimens have a tiny impact of water aging on flexural strength, a 9.7 % decrease in pH 8 medium. Table 5 shows the summary of flexural strength by experimental and *FEM* of base and in pH 8 condition. Fig. 5, Fig. 6 illustrate the contour plots of the flexural simulation of a type-*D*(0°) specimen and the comparison of the flexural modulus of base samples and samples immersed in a pH 8 solution, respectively. Type-*C*(0°) has superior bending modulus among the rest of the types, even after aging. The outside carbon layers (*C*/*J*/*J*/*C*), provide superior bending quality from the carbon fibers [30,39].

**Table 5:** Summary of flexural strength of base samples and in pH 8 condition.

Composite Type	*σ*_u_ (Expt.) Base (MPa)	*σ*_u_ (*FEM*) Base (MPa)	Deviation (%)	*σ*_u_ (Expt.) pH8 (MPa)	*σ*_u_ (*FEM)* pH8 (MPa)	Deviation (%)
*A*(0°)	83.6	83.60	0	68.6	68.6	0
*A*(45°)	28.5	28.5	0	36.3	36.1	0.55
*A*(90°)	12.0	12.0	0	11.46	11.31	1.31
*B*(0°)	89.1	89.1	0	88.5	88.5	0
*C*(0°)	299	295.35	1.22	270	270	0
*D*(0°)	317	317	0	228	228	0

The simulation discovered a little deviated flexural stress than the experimental approach. It is because the laminates were considered homogeneous during modeling, and there are no voids or cracks in the resulting composites. During an experimental flexural test, the laminates' non-homogeneous nature caused voids and a fiber-matrix break to appear [4,[32], [33], [34]]. Moreover, interfacial bonding is also a reason for the deviation between the numerical and experimental results [40].

### 3.4 *SEM* analysis

**Fig. 7:**
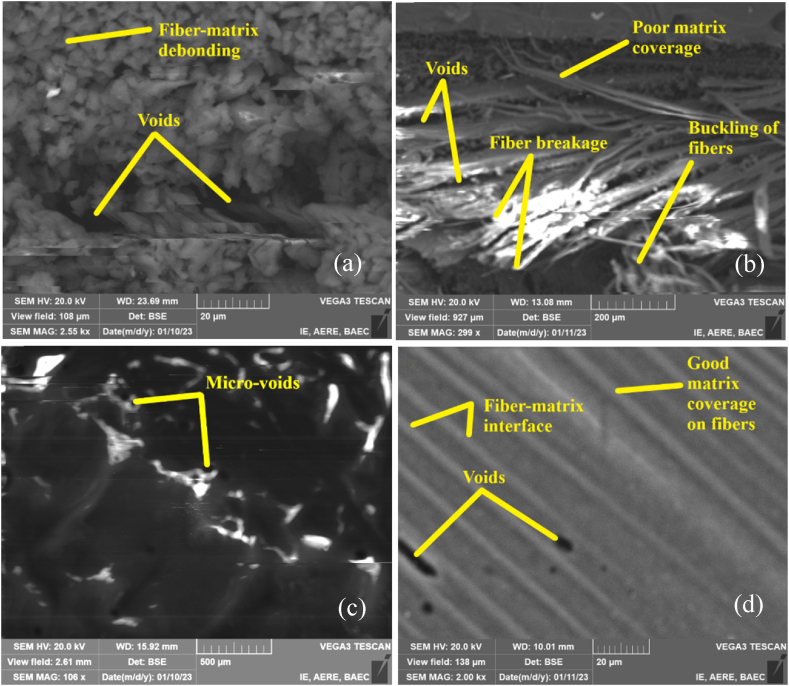
Micrographs of the composite sample pre-immersion (base) by Scanning Electron Microscopy (*SEM*): (a) Type-*A,* (b) Type-*B*, (c) Type-*C,* (d) Type-*D.*

Fig. 7(a–d) illustrates the *SEM* micrographs of jute and jute/carbon hybrid composites of base samples. The composite plate shows fiber-matrix de-bonding, fiber breakage, fiber buckling, and voids. Very tiny jute fiber-resin debonding was detected in type-*A* composites, which indicates that the cohesion between the fiber and matrix is excellent. In addition, fewer voids and fiber matrix interface gaps are seen in the fractographic assessment. In type-*B (J/C/C/J)*, voids, poor matrix coverage, fiber breakage, and buckling of fiber are observed. Buckling happens when a fiber experiences high compressive stresses and fails to collapse under the load, causing localized material deformation and distortion [[41], [42], [43]]. Type-*B* having higher amounts of defects, as observed from the *SEM*, performs poorly on flexural strengths. Which were also observed in prior works for other types of hybrid composites [34,43,44]. In type-*C*, and type-*D*, micro-voids and fiber-matrix de-bonding are found. Fiber-matrix debonding occurs when the material is subjected to mechanical or thermal stresses and the stress concentration at the fiber-matrix interface is greater than the bond strength between the fiber and the matrix [45]. The de-bonding of fibers from the matrix decreases the composite material's effective stiffness and strength and causes voids and delamination in the structure [46]. Good matrix coverage is seen in type-*D* composites. As the fiber and matrix have different mechanical characteristics and thermal expansion coefficients, this process results in stress concentrations at the interface of the two materials. The stiffness, strength, and thermal expansion coefficients of the carbon and jute fibers in carbon/jute hybrid composites differ from those of the epoxy matrix [30].

**Fig. 8:**
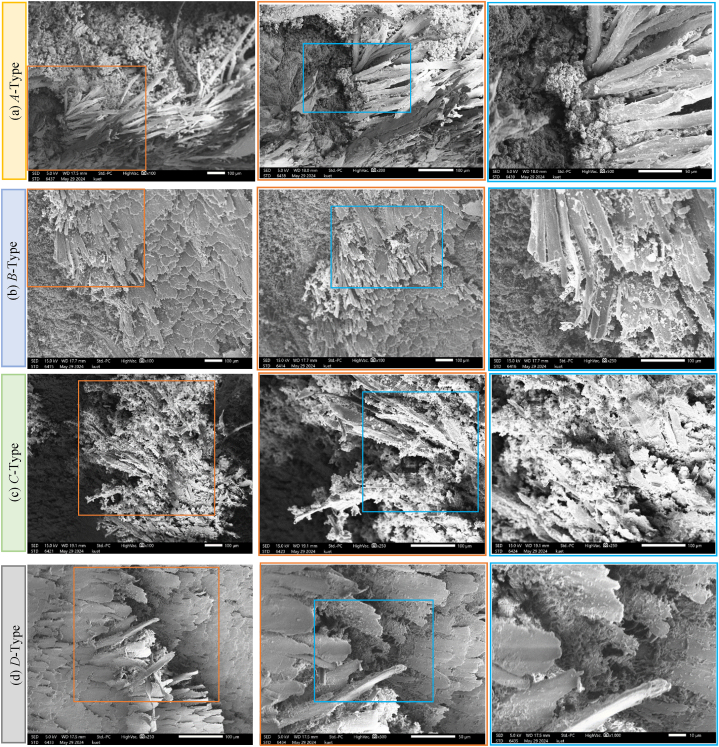
Micrographs of the composite sample post-immersion (pH 8) by Scanning Electron Microscopy (*SEM*): (a) Type-*A,* (b) Type-*B*, (c) Type-*C,* (d) Type-*D.*

Fig. 8(a–d) illustrates the *SEM* images of the composite samples after being immerged in a NaCl solution for about 45 days. Fig. 8 illustrates the post-immersion fractographic assessment of the composites by *SEM.* Type *A*(0°) having only jute fibers as reinforcements shows cylindrical jute fibers, alongside voids and globular salt deposition on fiber-matrix interfaces. While Type-*C*(0°) had carbon fibers as reinforcements alongside jute fibers, the *SEM* images highlighted the carbon fibers surrounded by NaCl deposition. Type-*B*(0°) and Type-*D*(0°) show carbon fiber pullout and matrix debonding. Due to the enhanced properties of carbon fibers, they tend not to break easily but rather cause pullout in the loading direction. The *SEM* images also show a small amount of matrix debris surrounding some carbon fibers. From Fig. 8, it was clearly shown that salt deposition is higher in jute fibers compared with carbon fiber. Therefore, the variation of tensile and flexural strength in the base and post-immersion conditions is higher in *A* type composites. In another study, it was shown that due to the deposition of NaCl at the fiber-matrix interface, the matrix cannot properly transfer the force among fibers [47]. Which explains the reduced amount of tensile and flexural behavior after the samples were immerged in NaCl solution [48]. The least amount of NaCl deposition on Type-*C*(0°) and Type-*D*(0°) from *SEM* images confirms their superior tensile and flexural properties even after immersion.

### 3.5 Impact of water absorption

**Fig. 9:**
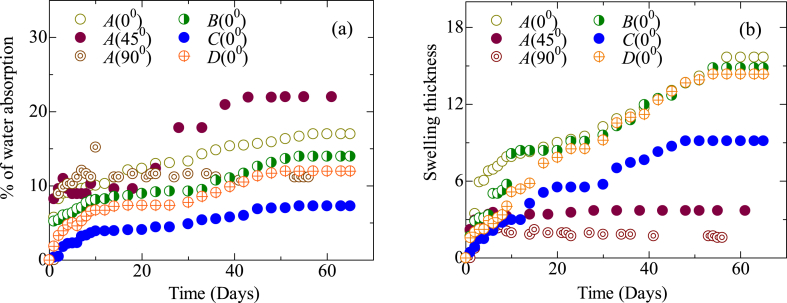
Moisture absorption behavior at pH 8: (a) Water absorption, (b) Swelling thickness.

According to Fig. 9(a), each composite laminate absorbs water very quickly within the first ten days before slowing down and eventually reaching a saturation level. As jute shows the hydrophilic property, the absorption of water is the highest in all type-*A* composites. In *B* type, where the sequence is *J/C/C/J*, the outer layer is jute, so the water is absorbed by the jute fiber. However, as carbon fibers show impermeability to moisture due to being hydrophobic, the water molecules hardly reach the core of the hybrid composite. Similar results were found in other synthetic fibers [7]. As a result, the water absorption and swelling thickness, which are shown in Fig. 9(a) and (b), respectively, are lower than in *B, C,* and *D* type composites. *C*-type (*C/J/J/C*) shows higher water resistance than the others as the exposed surface to water is both carbons. Synthetic fibers are hydrophobic in nature, repelling water absorption more than natural fiber [7,49,50]. Water molecules begin to move through the micro-cracks in the matrix as soon as they begin to crack both the *B*, *C,* and *D* type hybrid composites. The high cellulose concentration of jute fiber (60–70 %) adds to more water getting into the interface via micro-fissures caused by fiber swelling, resulting in swelling stresses and composite failure. Capillarity and transport through micro fissures become active as the composite cracks and is degraded [50,51]. Finally, capillary action caused by water molecules attacking fiber-matrix interfaces causes de-bonding between fibers and matrix to begin. The capillarity mechanism involves the movement of water molecules at fiber-matrix contacts as well as a diffusion process across the bulk matrix [[49], [50], [51], [52]].

### 3.6 Kinetics of diffusion mechanism

The diffusion mechanism is a primary contributor to the water absorption behavior of composite materials. Fick's theory is commonly used to model this mechanism and determine the kinetics of the absorption process. This approach has been previously applied to analyze the water absorption behavior of cellulose fiber-reinforced composites [26,27,[53], [54], [55]]. In the field of polymer diffusion, three distinct types of diffusion behavior have been identified based on the relative rates of diffusion and relaxation processes. The first type, known as Fickian diffusion or Case I, occurs when the rate of diffusion is much slower than the rate of polymer segment mobility. Equilibrium is quickly established and maintained independently of time. The second type of diffusion, known as Case II and Super Case II, occurs when the mobility of the diffusing species is much higher than other relaxation processes. In these cases, a boundary is formed between the swollen outer layer and the inner glassy core of the polymer, and this boundary moves at a constant velocity until an equilibrium concentration is reached throughout the polymer. Finally, Case III, or non-Fickian diffusion, occurs when the mobility of the diffusing species is comparable to the relaxation processes of the polymer segments. These three processes may be identified theoretically by the form of the absorption curve, which can be represented by the following Eq. (8) [25,26,56].

(8) $$\frac{M_{t}}{M_{m}}=kt^{n}$$

where *M*_t_ represents the moisture content at time *t* from Eq. (1), *M*_m_ represents the moisture content at saturation, on the contrary, *k* and *n* are constants. *k* is a constant that represents the interaction between the sample and the moisture, whereas, *n* represents the mode of diffusion. The value of *n*, in specific, differs across the three circumstances. For Fickian diffusion (Case I), *n* = 0.5 for Case II, *n* = 1 (and for Super Case II *n* > 1); and for Case III (Anomalous diffusion), 0.5 < *n* < 1. According to Eq. (9), which is derived from Eq. (8), the values of *n* and *k* were calculated from the slope and intercept of *M*_t_/*M*_m_ versus *t* in a log plot derived from the experimental data [25,26].

(9) $$\log\left(\frac{M_{t}}{M_{m}}\right)=\log\left(k\right)+n\;\log\left(t\right)$$

**Fig. 10:**
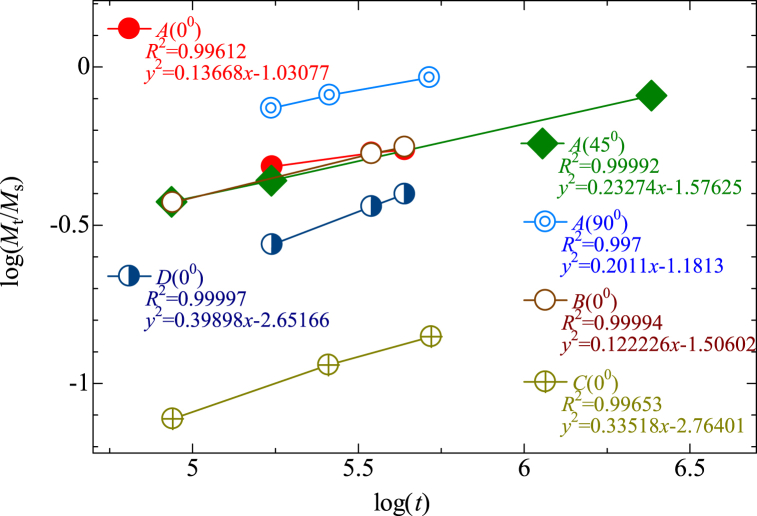
log (*M*_t_/*M*_m_) vs. log (*t*) curve of fabricated composites immersed in seawater (pH 8): (a) *A*(0°) type, (b) *A*(45°) type, (c) *A*(90°) type, (d) *B*(0°) type, (e) *C*(0°) type, (f) *D*(0°) type.

Fig. 10 illustrates the log (*M*_t_/*M*_m_) vs. log (*t*) curve of fabricated composites immersed in seawater (pH 8). Table 6 shows the values of the diffusion coefficient and its parameters. The results illustrated that type *A, B,* and *C* composites follow Fickian diffusion (Case I), suggesting a distinct diffusion behavior. Fiber swelling, fiber-matrix interface deterioration, micro-cracking, and leaching are all processes that lead to an overall moisture absorption in fiber-reinforced composites. This is a frequent phenomenon that has previously been seen in natural fiber-reinforced polymers [25,26,52,54,55,57]. According to Fick's model, the diffusion coefficient (*D*) is the most crucial parameter, representing the ability of water molecules to permeate composites. The obtained values are greatly influenced by the type and amount of fiber present in the composites. Specifically, type *D* composites exhibit the highest value (*n*) among them. When carbon fibers are introduced, the coefficient of diffusion tends to decrease. However, direct comparison of coefficient values with those obtained in other studies is challenging due to the dependence on materials, manufacturing methods, and test conditions [28,57]. Abid et al. found similar types of results with jute/Kevlar hybrid composites [25].

**Table 6:** Water absorption parameters for fabricated jute/carbon composites.

Composite type	*D* (m^2^/s)	*D*_c_(m^2^/s)	*n*	*K (h*^*-n*^*)*
*A* (0°)	7.65 x 10^−12^	2.33 x 10^−12^	0.137	1.031
*A* (45°)	8.71 x 10^−12^	2.657 x 10^−12^	0.233	1.58
*A* (90°)	3.4 x 10^−11^	1.037 x 10^−11^	0.201	1.18
*B* (0°)	1.605 x 10^−12^	7.977 x 10^−13^	0.222	1.5060
*C* (0°)	1.452 x 10^−12^	6.708 x 10^−13^	0.335	2.7640
*D* (0°)	1.51 x 10^−12^	7.218 x 10^−13^	0.398	2.6516

### 3.7 Density and void content

Table 7 illustrates the void fraction rates of the manufactured composites and hybrids. Notably, both Type-*A*(0°) and Type-*C*(0°) exhibit significantly elevated void fraction rates, which could potentially be attributed to imperfections in the manufacturing process. The percentage of void fraction is found to be highest in type-*A*(0°), which is 9.48 %, and lowest in type-*A*(90°), which is 1.73 %.The uneven distribution of the resin-hardener solution may be a result of inadequate adhesion between the fibers and the matrix. Furthermore, Fig. 9(a) reveals that Type-*A* composites display noticeably higher rates of water absorption. This could be indicative of water seeping into the voided areas during the immersion process [25]. Voids have a relatively minor impact on the fiber-dominant characteristics of composites. However, it's worth noting that properties primarily dependent on the matrix, like interlaminar shear strength (ILSS), are significantly affected by the presence of voids [44,58,59]. The mechanical properties of composites decrease with increasing void fraction, and the shape of the voids also affects their tensile properties [35].

**Table 7:** Void fraction rate (*V*_*v*_) of fabricated composites and hybrids.

Type	*ρ*_t_ (gm/cm^3^)	*ρ*_a_ (gm/cm^3^)	*V*_*v*_(%)
*A* (0°)	1.16	1.05	9.48
*A* (45°)	1.16	1.07	7.76
*A* (90°)	1.16	1.14	1.73
*B* (0°)	1.206	1.154	4.31
*C* (0°)	1.202	1.099	8.57
*D* (0°)	1.203	1.144	4.91

## 4 Conclusion

The hybridization of carbon fibers with jute fibers essentially enhanced the mechanical properties of the composites. The stacking sequence played a pivotal role in determining both mechanical and water absorption properties, particularly depending on whether the outer surface consists of carbon or jute mat. *FEM* analysis validated the tensile and flexural behaviors of the composites, while highlighting differences in flexural strain due to variations in homogeneity. Type-*C*(0°) configurations demonstrated superior properties in both tensile and flexural strength compared to Type-*A*(0°). The introduction of carbon fibers in Type-*C*(0°) composites resulted in a remarkable 361 % increase in tensile strength and a 258 % increase in flexural strength compared to that of Type-*A*(0°). Notably, Type-*D*(0°) also displayed commendable performance. Higher flexural strength was exhibited when carbon fiber was placed on the outer surface (the compression side of the samples during testing) of the hybrid composites (type-*C* and type-*D*). Upon aging in corrosive mediums, Type-*C*(0°) exhibited a 40 % decrease in tensile strength and a 9.7 % decrease in flexural strength. The results indicated the superior performance of Type-*C*(0°) compared to Type-*D*(0°). The bending capability of Type-*C*(0°) surpassed that of Type-*D*(0°). Despite having a higher void percentage for Type-*C*(0°) than Type-*D*(0°), Type-*C*(0°) still performed better. Moreover, swelling thickness and water absorption percentage were considerably lower in Type-*C*(0°) compared to other types, contributing to enhanced tensile and flexural performance even after aging in corrosive mediums. However, the salt deposition and water penetration in the composites are the major causes of strength degradation when the composites are immersed in corrosive mediums. It's worth noting that composites and hybrids containing natural fibers absorb water through Fickian diffusion, with micro-crack formation and matrix erosion being major contributors to mechanical degradation.

Therefore, the results clearly made type *C*(0°) highly suitable for high-performance composites while maintaining the attractive appearance of traditional *CFRP* on both surfaces.

## Data availability statement

Data will be available on request.

## CRediT authorship contribution statement

**Md Sadbin Azad Alvy:** Writing – original draft, Investigation. **Md Foisal Hossain:** Writing – review & editing, Resources, Project administration. **Muhammed Sohel Rana:** Writing – review & editing, Software. **Md Motinur Rahman:** Investigation. **Md Shafiul Ferdous:** Writing – review & editing, Methodology, Formal analysis, Conceptualization.

## Declaration of competing interest

The authors declare that they have no known competing financial interests or personal relationships that could have appeared to influence the work reported in this paper.
